# Faculty members of Islamic Azad University of Zanjan's knowledge regarding AIDS and preventing it

**DOI:** 10.1186/1742-4690-9-S1-P127

**Published:** 2012-05-25

**Authors:** Vida Sadeghzadeh, Eshrat Sadeghzadeh

**Affiliations:** 1 University Department of Nursing, Zanjan Branch, Islamic Azad University, Zanjan, Iran

## Introduction

According to UNAIDS estimates, there are now 33.3 million people living with HIV, including 2.5 million children. During 2009 some 2.6 million people became newly infected with the virus and an estimated 1.8 million people died from AIDS. By mid 2011, 23125 people were infected with HIV, from which, 4311 people died in Iran. The vast majority of people with HIV and AIDS live in lower- and middle-income countries. But HIV today is a threat to men, women and children on all continents around the world. Since there is no vaccine for AIDS and there is no certain cure for AIDS, curing, prevention and secondary infection is essential. This study was conducted to determine the level of knowledge on AIDS and the way of preventing it among faculty members of Zanjan branch, Islamic Azad University.

## Materials and methods

This descriptive study involves 90 people. Data was collected by a questionnaire in five parts (Demographic questions, factors related to transmission, pathology, complications, and finally prevention of AIDS). Analysing of data was conducted by SPSS software.

## Results

Findings revealed that awareness of faculty members of Islamic Azad University of Zanjan about transmission of disease was 72.2, regarding pathology of disease was 67.7, related to complication of disease was 73.3, and about prevention of disease was 74.4.Finally, the level of knowledge of faculty members of Islamic Azad University of Zanjan was higher than moderate (average %70).

## Conclusion

It can be concluded that the rate of awareness is almost satisfactory but it is not enough and it is necessary to perform educational programs. Key Words: AIDS, Faculty member, Knowledge, Prevention.

